# Anti-angiogenesis in human lymphomas. New insights

**DOI:** 10.3389/fonc.2025.1636332

**Published:** 2025-10-08

**Authors:** Domenico Ribatti, Roberto Tamma, Giuseppe Ingravallo, Giorgina Specchia

**Affiliations:** ^1^ Department of Translational Biomedicine and Neuroscience, University of Bari Medical School, Bari, Italy; ^2^ Department of Precision and Regenerative Medicine and Ionian Area, University of Bari Medical School, Bari, Italy

**Keywords:** angiogenesis, anti-angiogenesis, Hodgkin lymphomas, non-Hodgkin lymphomas, tumor progression

## Abstract

Targeting tumor angiogenesis is an advancement in the treatment of hematological malignancies. This article summarizes the most recent advancements in the use of anti-angiogenic agents in the treatment of human lymphomas. Preclinical and clinical studies have evidenced that treating lymphomas with anti-angiogenic monotherapy may not be successful. Alternative therapeutic strategies may be used to overcome resistance to anti-angiogenic therapy, including the association of different anti-angiogenic molecules or their combination with other treatment regimens.

## Introduction

Anti-angiogenesis was proposed as a cancer therapy in 1971, when J. Folkman published a hypothesis that tumor growth is angiogenesis-dependent (1). Anti-angiogenic agents include anti- vascular endothelial growth factor (VEGF) molecules agents, including bevacizumab, VEGF-Trap, and VEGF-antisense; receptor tyrosine kinase inhibitors (TKIs);immunomodulatory drugs (iMiDs), including thalidomide, lenalidomide, and pamalidomide; other new compounds targeting signaling checkpoints downstream of pro-angiogenic growth factors, including mammalian target of rapamycin (mTOR) inhibitors, histone deacetylases (HDAC) inhibitors, and proteasome inhibitors, such as bortezomib and carfilzomib.

It is important to note that among these agents, iMiDs and proteasome inhibitors are primarily known for other mechanisms of action. In detail, iMiDs work by either enhancing or suppressing the immune system, with specific mechanisms varying by drug type. They can degrade target proteins within tumor cells and activate immune cells like T cell and NK cells, leading to tumor cell death and a stronger immune response. Other immunomodulators, such as checkpoint inhibitors, block proteins that suppress immune responses, while others, like colony-stimulating factors, stimulate the production of immune cells. Proteasome inhibitors block the 20S proteasome, a complex that degrades damaged or unneeded proteins, causing an accumulation of misfolded proteins within the cell, leading to cellular stress, ER stress, and the eventual activation of apoptotic pathways. In this context the anti-angiogenic activity of these molecules may be considered expression of a secondary mechanism of action.

This article summarizes the most recent advancements concerning anti-angiogenic agents in the treatment of human lymphomas.

## Non-Hodgkin lymphomas

### Monotherapy trials

Bevacizumab has shown modest clinical activity in lymphoma patients as a single agent in the setting of relapsed aggressive non-Hodgkin lymphoma (NHL) when administered at 10 mg/kg every 2 weeks in a Southwest Oncology group (SWOG) phase II trial (2, 3).

As concerns sorafenib, in phase II study, 30 patients with relapsed lymphoma received sorafenib 400 mg twice daily for a median of 4 months (4). Despite a disappointing 13% overall response rate (ORR), sorafenib was well tolerated and produced some degree of clinical activity. The low efficacy of sorafenib as a single agent was also reported in phase II study with 14 relapsed diffuse large B cell lymphoma (DLBCL) patients who failed or were not candidates for autologous stem cell transplant, as shown by only 1 patient had complete response (CR) and 13 patients died (5). Brander et al. (6) conducted a phase II open-label study to assess the efficacy and safety of vatalanib in relapsed or refractory DLBCL patients who received it at a target dose of 1250 mg once daily. Imatinib, a impairs lymphoma growth in both human xenograft and murine allograft models by inhibiting tumor-associated angiogenesis (7).

Single-agent thalidomide demonstrated a limited and modest overall response rate of 12.5% when given to patients with relapsed/refractory indolent NHL (8). One patient with gastric mucosa-associated lymphoid tissue (MALT) lymphoma achieved complete response 2 months after initiation of thalidomide, which was accompanied with significantly decreased serum levels of VEGF-A and fibroblast growth factor-2 (FGF-2) (9).

Single agent IMiD lenalidomide has been studied in a phase II trial setting in relapsed/refractory indolent and aggressive NHL, with a 34% ORR rate in aggressive NHL and a 26% ORR rate in in indolent NHL (10–12). Three phase II studies (13–15) reported that single-agent lenalidomide was active and safe in both indolent and aggressive B-NHL. In DLBCL, lenalidomide exhibited preferential activities in non-germinal center B-cell-like (non-GCB) subtype than in GCB subtype (16). A phase II study demonstrated a better response of lenalidomide in patients with relapsed or refractory mantle cell lymphoma (MCL) when compared to control therapies such as rituximab, gemcitabine, fludarabine, chlorambucil, or cytarabine (17). Treatment with the immunomodulatory lenalidomide, depleted VEGF-C expressing tumor associated macrophages (TAMs) resulted in impaired lymphangiogenesis in MCL (18).

Two fully humanized IgG4-kappa immune checkpoint inhibitors blocking monoclonal antibodies targeting the PD-1 receptor on human T cells, nivolumab and pembrolizumab and three anti-PD-L1 antibodies (durvalumab, atezolizumab, and avelumab) have been approved for the treatment of B cell lymphomas (19, 20). Follicular lymphoma (FL) and DLBCL presented the highest objective response to therapy with nivolumab, while MCL lacked a response to treatment (21). In DLBCL treatment with nivolumab, a phase I study demonstrated an ORR of 36% (21), and a phase II study with the same treatment an objective RR of 3% (22).

### Combination therapy trials

Bevacizumab has been combined with rituximab- CHOP (R-CHOP) in upfront treatment (23). The combination of bevacizumab and standard-dose R-CHOP (RA-CHOP) did not improve the survival time of untreated DLBCL and MCL patients (24, 25). Moreover, the addition of bevacizumab to R-CHOP in a phase II DLBCL trial increased cardiac events without significantly improving efficacy (26).

A significantly improved progression free survival (PFS) was observed in patients who received rituximab plus bevacizumab compared to those who received rituximab alone in relapsed FL (27). Overall survival (OS) was also prolonged numerically but did not reach statistical significance. Both regimens were well tolerated even though the addition of bevacizumab did increase side effects (27).

Thalidomide has indicated therapeutic potential in patients with relapsed MCL, either as a single agent (28) or combined with chemotherapy (29). Thalidomide enhances the efficacy of CHOP in treating patients with DLBCL. Compared to patients treated by CHOP alone, although there was no significant OS difference between the two groups, the median PFS in T-CHOP group was significantly prolonged (30).

The generalized effect of lenalidomide plus rituximab was highly active, achieving a 92% ORR and a 64% CR in untreated MCL patients (31) and a 57% ORR and a 36% CR in relapsed or refractory MCL patients (32).

A combination of rituximab, thalidomide, and metronomic oral chemotherapy with prednisone, etoposide, procarbazine, and cyclophosphamide have been used as anti-angiogenic therapy in relapsed/refractory MCL (33). Another metronomic lymphoma therapy is the Prednisone, etoposide, procarbazine, and cyclophosphamide (PEPC) (C3) regimen (34). This regimen is well tolerated and is associated with significant clinical activity in the recurrent NHL including MCL (35, 36).

Durvalumab administration in combination with rituximab and bendamustine was associated with an ORR of 88.9% in FL, and of 30% in DLBCL (37). Combination therapy with durvalumab and ibrutinib, a Bruton’s tyrosine kinase (BKT) inhibitor, was associated with an ORR in MCL (37). Atezolizumab in combination with R-CHOP in patients with previously untreated DLBCL improves complete remission rates compared with controls (38). Otherwise, a favorable clinical activity has been reported when a PD-1 inhibitor was administrated with an anti-CD20 monoclonal antibodies in RR B-cell lymphomas (39). Dual checkpoint blockades with anti-PD-1 and CTLA-4 monoclonal antibodies, with or without STAT3 inhibitors, did not show clinical activity in DLBCL and FL (40).

## Hodgkin lymphomas

### Monotherapy trials

Pembrolizumab, that blocks the PD-1/PD-L1 and PD-2/PDL2 pathway, showed positive survival outcomes in patients with RR cHL (41). Tislelizumab, a humanized anti PD-1 monoclonal antibody, demonstrated a favorable safety profile for patients with RR cHL (42).

### Combination therapy trials

Sorafenib and perifosine (an oral Akt inhibitor) combination therapy demonstrated antitumor activities in HL patients (43). The efficacy and safety of nivolumab have been investigated in RR classic HL (cHL), demonstrating an ORR of 87% and a progression-free survival of 86% in 24 weeks (44).

The combination of the immune checkpoint inhibitor nivolumab with doxorubicin, vinblastine, and dacarbazine (AVD) was evaluated in patients with stage III and IV cHL, showing an objective RR of 84% with complete remission in 67% of patients (45). Nivolumab-AVD versus brentuximab-AVD was studied in advanced stage cHL, demonstrating an increase in PFS with nivolumab-AVD group compared to the brentuximab-AVD group (46). Pembrolizumab and brentuximab have been compared vedotin in patients with RR cHL, demonstrating that the two-year PFS and safety outcomes favored the use of pembrolizumab (47). It is important to note that the primary mechanism of action of both nivolumab and pembrolizumab is not anti-angiogenesis, but their effectiveness is due to a separate, through potentially synergistic, immune-mediated process.

## Comparative efficacy, toxicity and mechanisms of resistance

Anti-angiogenic therapy with bevacizumab has modest activity in DLBCL. The combination of bevacizumab and R-CHOP has been investigated in both DLBCL and MCL. iMiDs, either as single agent or in combination with fludarabine or rituximab, have clinical activity against a wide variety of NHL including DLBCL, CLL and MCL. Metronomic low-dose chemotherapy has clinical applicability in in relapsed and refractory settings. Biological agents, targeting RTKs, are in various stages of clinical development and investigation in human lymphoma patients.

As concerns resistance mechanisms, when VEGF-targeted therapies are discontinued, the tumor vasculature is rapidly re-established (48). Resistance to anti-angiogenic therapies can be intrinsic, when it is observed at the beginning of the treatment due to inefficacy of treatment or acquired, i.e., that it affects the relapsing disease after an initial response to therapy (49). Resistance mechanisms are mediated by hypoxia. VEGF blockade aggravates hypoxia that, in turn, upregulates the production of angiogenic factors or increases tumor invasiveness (49); redundancy of the angiogenic signals, and activation of alternative signaling pathways (50); Upregulation of other pro-angiogenic factors, including FGF-2 and platelet derived growth factor (PDGF) (50).

Side effects of anti-angiogenic agents of lymphoma treatment include hypertension, bleeding, impaired wound healing, and proteinuria (51–53). While not directly a “side effect” of the anti-angiogenic mechanism, these drugs can also increase the risk of neutropenia when combined with traditional chemotherapy, highlighting potential overlapping toxicities. Anti-angiogenic agents share common side effects, including hypertension, proteinuria, impaired wound healing, and bleeding, though the frequency and severity vary between specific drugs. Other common adverse events can include thrombosis fatigue, diarrhea, and hand-foot syndrome. For example, bevacizumab has a higher incidence of bleeding complications, while small-molecule VEGF receptors (VEGFRs) inhibitors are associated with hand-foot syndrome and elevated liver enzymes.

## Concluding remarks

Treating human lymphomas with anti-angiogenic monotherapy is not successful. The limitations of may be the effect of drug resistance, metastasis promotion, and reduced delivery of chemotherapeutic agents, because of the decrease in tumor vasculature.

Different anti-angiogenic biomarkers have been introduced, including changes in systemic blood pressure, VEGF polymorphisms, plasma levels of VEGF, tumor microvascular density and imaging parameters. The dose and schedule of anti-angiogenic and cytotoxic therapies when used in combination might not be optimal, and biomarkers could be useful in optimizing the dose and schedule of these agents.

The fact that tumors may grow without angiogenesis, through the alternative mode of vasculature neo-formation, including vascular co-option, intussusceptive microvascular growth, and vasculogenic mimicry (54) makes them less likely to respond to anti-angiogenic drugs. Crivellato et al. (55) demonstrated that in B-cell NHLs at the ultrastructural level, tumor cells are closely intermingled with endothelial cells and that this relationship can be recognized in the early stages of vessel formation, as an expression of vasculogenic mimicry. Moreover, Crivellato et al. (55) showed that both low- and high-grade B-NHLs develop transluminal bridges in larger vessels, causing the parent vessel to split into two or more sections, suggesting that an intussusceptive modality of vascular growth also takes place in B-NHLs.

Other therapeutic strategies may be used to overcome resistance to anti-angiogenic therapy, including the association of multiple anti-angiogenic compounds or a combination of anti-angiogenic drugs with other treatment regimens, such as immunotherapies to enhance antitumor immune responses has proven to be an effective approach. Combining anti-VEGF agents with immunotherapies an established and effective cancer treatment strategy that has improved outcomes for patients with several types of cancer, including non-small cell lung cancer, renal cell carcinoma, and hepatocellular carcinoma. This combination therapy works by not only directly targeting cancer cells but also by decreasing the immunosuppressive effects of VEGF, thereby allowing the immune system to better attack the tumor. Combining anti-VEGF agents with immunotherapies offers several advantages, including normalizing tumor blood vessels to improve immune cell infiltration, enhancing antigen presentation by maturing dendritic cells, and reducing the presence of immunosuppressive cells. This synergy leads to improved clinical outcomes, such as OS and PFS (56, 57).
